# Non-iatrogenic esophageal injury: a retrospective analysis from the National Trauma Data Bank

**DOI:** 10.1186/s13017-017-0131-8

**Published:** 2017-04-27

**Authors:** Alberto Aiolfi, Kenji Inaba, Gustavo Recinos, Desmond Khor, Elizabeth R. Benjamin, Lydia Lam, Aaron Strumwasser, Emanuele Asti, Luigi Bonavina, Demetrios Demetriades

**Affiliations:** 1 Department of Biomedical Sciences for Health, University of Milan, IRCCS Policlinico San Donato, Piazza Edmondo Malan, 1, 20097 Milan, Italy; 2 Division of Trauma and Surgical Critical Care, LAC+USC Medical Center, University of Southern California, 2051 Marengo Street, Los Angeles, CA 90033 USA

**Keywords:** Esophageal trauma, Non-iatrogenic esophageal injury, Primary suture, Outcomes

## Abstract

**Background:**

Traumatic, non-iatrogenic esophageal injuries, despite their rarity, are associated with significant morbidity and mortality. The optimal management of these esophageal perforations remains largely debated. To date, only a few small case series are available with contrasting results. The purpose of this study was to examine a large contemporary experience with traumatic esophageal injury management and to analyze risk factors associated with mortality.

**Methods:**

This National Trauma Data Bank (NTDB) database study included patients with non-iatrogenic esophageal injuries. Variables abstracted were demographics, comorbidities, mechanism of injury, Abbreviated Injury Scale (AIS), esophageal Organ Injury Scale (OIS), Injury Severity Score (ISS), level of injury, vital signs, and treatment. Multivariate analysis was used to identify independent predictors for mortality and overall complications.

**Results:**

A total of 944 patients with non-iatrogenic esophageal injury were included in the final analysis. The cervical segment of the esophagus was injured in 331 (35%) patients. The unadjusted 24-h mortality (8.2 vs. 14%, *p* = 0.008), 30-day mortality (4.2 vs. 9.3%, *p* = 0.005), and overall mortality (7.9 vs. 13.5%, *p* = 0.009) were significantly lower in the group of patients with a cervical injury. The overall complication rate was also lower in the cervical group (19.8 vs. 27.1%, *p* = 0.024). Multilogistic regression analysis identified age >50, thoracic injury, high-grade esophageal injury (OIS IV–V), hypotension on admission, and GCS <9 as independent risk factors associated with increased mortality. Treatment within the first 24 h was found to be protective (OR 0.284; 95% CI, 0.148–0.546; *p* < 0.001). Injury to the thoracic esophagus was also an independent risk factor for overall complications (OR 1.637; 95% CI, 1.06–2.53; *p* = 0.026).

**Conclusions:**

Despite improvements in surgical technique and critical care support, the overall mortality for traumatic esophageal injury remains high. The presence of a thoracic esophageal injury and extensive esophageal damage are the major independent risk factors for mortality. Early surgical treatment, within the first 24 h of admission, is associated with improved survival.

**Trial registration:**

iStar, HS-16-00883

## Background

The management of iatrogenic and spontaneous perforations of the esophagus has well-established risk factors and treatment guidelines. In this setting, thoracic perforations are associated with poor outcomes because of the association with systemic sepsis and multi-organ failure [1–5]. In contrast, small and well-contained cervical perforations are associated with better outcomes [6]. Prompt diagnosis and early treatment have been shown to improve outcomes [7–9].

Despite its rarity, traumatic esophageal injury is associated with a significant morbidity and mortality burden. To date, only a few small series are available in the literature addressing management and outcomes with contrasting results. As a result, our current understanding of the optimal treatment for these injuries is unclear.

The purpose of this study was to examine a large contemporary experience with traumatic esophageal injury management, to compare cervical and thoracic injury, and to analyze risk factors associated with mortality.

## Methods

After Institutional Review Board approval, the National Trauma Data Bank (NTDB) was queried to identify all patients 16 years and older who sustained a traumatic esophageal injury (ICD-9 codes 862.22 and 862.32) over a 7-year period (2007–2014). Patients transferred from an outside hospital and those who died upon arrival were excluded from the study. Spontaneous (Boerhaave syndrome) and iatrogenic esophageal perforations that occurred during upper gastrointestinal endoscopy were also excluded from the final analysis.

Variables extracted from the NTDB included demographics, comorbidities, mechanism of injury, Abbreviated Injury Scale (AIS), Injury Severity Score (ISS), Organ Injury Scale (OIS), and vital signs in the emergency department. The location of the esophageal injury (cervical vs. thoracic), treatment modalities (primary repair vs. esophagectomy vs. esophagostomy), and timing of surgical treatment were abstracted. Outcomes of interest included in-hospital mortality, complications, ventilation days, ICU length of stay, and hospital length of stay.

The study population was further subdivided and analyzed by the level of esophageal injury: cervical or thoracic. Severe injury was defined as AIS 3 or higher in any body region. Early surgical treatment was defined as operative intervention performed in the first 24 h. Isolated esophageal injury was defined as an esophageal injury with no other associated injuries with an AIS ≥3.

### Statistical analysis

Categorical variables were reported as percentages, while continuous variables were reported as medians with interquartile range (IQR). Continuous variables were also dichotomized using clinically relevant cut-off points. Univariate analysis was performed to identify differences between outcomes in groups of interest. The Mann-Whitney *U* test was used to compare continuous variables while Fisher’s exact or Pearson’s chi-squared test was used to compare proportions for categorical variables. Variables with *p* < 0.2 in univariate analysis were included into a forward stepwise logistic regression to identify independent predictors for mortality and the development of complications. Multicollinearity testing was performed to identify the correlation between variables. The accuracy of the test is calculated using the area under the curve with a 95% confidence interval. Variables with a *p* value <0.05 were considered significant. All statistical analysis was performed using SPSS for Windows version 23.0 (SPSS Inc. Chicago, IL).

## Results

**Fig. 1:**
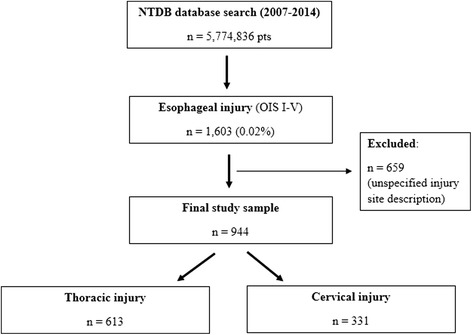
Study flow chart. *NTDB* National Trauma Data Bank, *OIS* Organ Injury Scale

During the study period, a total of 1603 patients were identified from the NTDB as having a traumatic esophageal injury, with an overall prevalence of 0.02% (1603/5,774,836). Due to an unspecified description of the esophageal injury, 659 patients (41.1%) were excluded from the final analysis leaving a final study population of 944 patients (Fig. 1).

### Demographics

Patients with an esophageal injury were more likely to be males (77.6%), with a median age of 35 years (IQR 24–52) and 27.4% were over 50 years of age. On admission, 9.4% of the cases were identified as being hypotensive (systolic blood pressure <90 mmHg), with a median heart rate of 97 (IQR 80–112), and a Glasgow Coma Scale (GCS) <9 was seen in 23.1% of cases.

### Mechanism of injury

Approximately half of the esophageal injuries were due to a penetrating injury mechanism (50.6%). Gunshot wounds, seen in 337 (35.7%) of patients, were the most common mechanism of injury for patients with a penetrating injury, followed by stab wounds in 14.9%. For patients sustaining blunt injuries, motor vehicle crash (MVC) was the most common mechanism of injury seen in 179 (19%) patients, followed by falls in 7.7%, and assault in 4.1% (Table 1).

**Table 1:** Demographics and clinical data according to the location of the esophageal injury

	Total	Thoracic	Cervical	
	(*n* = 944)	(*n* = 613)	(*n* = 331)	*p*
Demographics				
Age (years), median (IQR)	35 (24–52)	35 (24–53)	36 (25–49)	0.885
Age >50 year	259 (27.4)	180 (29.4)	79 (23.9)	0.071
Gender, male	731 (77.6)	469 (76.8)	262 (79.2)	0.4
Race/ethnicity				
White	534 (56.6)	342 (55.8)	192 (58)	0.512
Black	240 (25.4)	148 (24.1)	92 (27.8)	0.219
Mechanism				<0.001
Blunt	335 (35.5)	241 (39.3)	94 (28.4)	
Penetrating	478 (50.6)	278 (45.4)	200 (60.4)	
GSW	337 (35.7)	210 (34.3)	127 (38.4)	0.208
SW	141 (14.9)	68 (11.1)	73 (22.1)	<0.001
MVC	179 (19)	133 (21.7)	46 (13.9)	0.004
AVP	12 (1.3)	10 (1.6)	2 (0.6)	0.179
Fall	73 (7.7)	51 (8.3)	22 (6.6)	0.358
MCC	26 (2.8)	18 (2.9)	8 (2.4)	0.642
Assault	39 (4.1)	26 (4.2)	13 (3.9)	0.817
Comorbidities				
Current smoker	141 (14.9)	72 (11.7)	69 (20.8)	<0.001
Chronic renal failure	4 (0.4)	2 (0.3)	2 (0.6)	0.53
Diabetes mellitus	51 (5.4)	34 (5.5)	17 (5.1)	0.79
Myocardial infarction	6 (0.6)	6 (1)	0 (0)	0.096
Hypertension	149 (15.8)	96 (15.7)	53 (16)	0.888
Obesity	48 (5.1)	25 (4.1)	23 (6.9)	0.055
Respiratory disease	62 (6.6)	32 (5.2)	30 (9.1)	0.023
Cirrhosis	2 (0.2)	2 (0.3)	0 (0)	0.544
ED vitals				
SBP <90 mmHg	89 (9.4)	63 (10.3)	26 (7.9)	0.224
HR (bpm), median (IQR)	97 (80–112)	97 (80–114)	96 (80–109)	0.258
GCS score <9	218 (23.1)	139 (22.7)	79 (23.9)	0.678
Injury description				
ISS, median (IQR)	24 (16–33)	24 (16–34)	21 (16–29)	0.181
ISS >15	761 (80.6)	495 (80.8)	266 (80.4)	0.886
Associated injuries (AIS ≥3)				
Head	270 (28.6)	167 (27.2)	103 (31.1)	0.209
Chest	860 (91.1)	559 (91.2)	301 (90.9)	0.896
Abdomen	161 (17.1)	132 (21.5)	29 (8.8)	<0.001
Extremities	119 (12.6)	86 (14)	33 (10)	0.073
Isolated esophageal injury	218 (23.1)	133 (21.7)	85 (25.7)	0.166
Esophageal OIS				0.103
OIS I–II	410 (43.4)	280 (45.7)	130 (39.3)	
OIS III	466 (49.4)	287 (46.8)	179 (54.1)	
OIS IV–V	68 (7.2)	46 (7.5)	22 (6.6)	
Procedures				
Tracheostomy	199 (21.1)	113 (18.4)	86 (26)	0.007
Trachea repair	103 (10.9)	45 (7.3)	58 (17.5)	<0.001
Surgical treatment	345 (36.5)	208 (33.9)	137 (41.4)	0.023
Early treatment (≤24 h)	275 (29.1)	163 (26.6)	112 (33.8)	0.019

Values are presented as median (IQR) and *n* (%)

*GSW* gunshot wound, *SW* stab wound, *MVC* motor vehicle collision, *AVP* auto versus pedestrian, *MCC* motorcycle collision, *SBP* systolic blood pressure, *HR* heart rate, *GCS* Glasgow Coma Scale, *ISS* Injury Severity Score, *AIS* Abbreviated Injury Scale, *OIS* Organ Injury Scale, *IQR* interquartile range

### Injury description

Patients presenting to the emergency department with an esophageal injury had a median ISS of 24 (IQR 16–33), with 80.6% having an ISS >15. Associated severe head, chest, and abdominal injuries were documented in 28.6, 91.1, and 17.1% of patients, respectively.

Injury to the thoracic esophagus occurred in 64.9% of patients, and the remaining 35.1% had a cervical esophageal injury. Patients with a cervical injury were also more likely to have an associated tracheal injury requiring surgical repair and needing tracheostomy (17.5 vs. 7.3%, *p* < 0.001 and 26 vs. 18.4%, *p* = 0.007, respectively). High-grade injury with full-thickness perforation occurred in 56.5% of patients (OIS III: *n* = 466, 49.4%; OIS IV/V: *n* = 68, 7.2%); low-grade esophageal injury occurred in 43.4% of patients (OIS I/II: *n* = 410). Overall, 218 patients (23.1%) had an isolated esophageal injury, and half of these had a low-grade injury (OIS I/II: *n* = 120, 55%).

Cervical esophageal injuries were more likely to occur following a penetrating mechanism (60.4 vs. 45.4%, *p* < 0.001) (Table 1). Compared to thoracic injuries, cervical injuries were less frequent after MVC (13.9 vs. 21.7%, *p* = 0.004) and less likely to have associated severe abdominal trauma (8.8 vs. 21.5%, *p* < 0.001). No difference in the median ISS, systolic blood pressure, GCS, and ISS >15 were noted in the two study groups (Table 1).

### Outcomes

Overall, 345 (36.5%) patients went to the operating room for exploration and 275 (79.7%) had a surgical intervention within the first 24 h of admission. Primary suture repair was performed in 317 (91.9%) patients. Patients with a cervical injury were more likely to undergo a primary repair (37.8 vs. 31.3%, *p* = 0.045). A drainage procedure was performed in 160 (16.9%) patients, and an esophageal stent was placed in 11 (1.2%) patients (Table 2). In the remaining 425 (45%) patients, the treatment was either non-operative or unspecified. Esophageal resection and diversion were more likely to be performed in patients with a grade III, IV, and V esophageal injury (Table 3).

**Table 2:** Different operative strategies according to the location of the esophageal injury

	Thoracic (*n* = 613)	Cervical (*n* = 331)	*p*
Primary suture (*n* = 317)	192 (31.3)	125 (37.8)	0.045
Esophagectomy (*n* = 15)	8 (1.3)	7 (2.1)	0.342
Esophageal diversion/esophagostomy (*n* = 13)	6 (1)	7 (2.1)	0.24
Esophageal stent (*n* = 11)	7 (1.1)	4 (1.2)	1
Perivisceral drainage (*n* = 160)	115 (18.8)	45 (13.6)	0.044

Values are presented as *n* (%)

**Table 3:** Different operative strategies according to the esophageal Organ Injury Scale (OIS)

	OIS I–II (*n* = 410)	OIS III (*n* = 466)	OIS IV–V (*n* = 68)	*p*
Primary suture (*n* = 317)	71 (17.3)	220 (47.2)	26 (38.2)	<0.001
Esophagectomy (*n* = 15)	0 (0)	13 (2.8)	2 (2.9)	0.003
Esophageal diversion/esophagostomy (*n* = 13)	0 (0)	9 (1.9)	4 (5.9)	<0.001
Esophageal stent (*n* = 11)	2 (0.5%)	9 (1.9%)	0 (0)	0.116
Perivisceral drainage (*n* = 160)	77 (18.8)	70 (15)	13 (19.1)	0.296

Values are presented as *n* (%)

The overall mortality was significantly higher in patients who sustained a blunt esophageal injury compared to patients with a penetrating injury (18.8 vs. 9.8%, *p* < 0.001). Thoracic esophageal injury was associated with significantly higher overall (14 vs. 8.2%, *p* = 0.008), 24-h (9.3 vs. 4.2%, *p* = 0.005), and 30-day mortality (13.5 vs. 7.9%, *p* = 0.009). No significant differences were noted in terms of hospital length of stay, ICU length of stay, and ventilation days. Pneumonia was the most commonly reported complication with a trend toward a higher incidence in the thoracic group (9.5 vs. 5.8%, *p* = 0.072). Sepsis and pulmonary embolism were higher in patients who sustained a thoracic injury (4.8 vs. 1.1%, *p* = 0.006 and 2.5 vs. 0%, *p* = 0.008, respectively), and the overall complication rate was higher in the thoracic esophageal group (27.1 vs. 19.8%, *p* = 0.024) (Table 4).

**Table 4:** Outcome comparison between patients with a thoracic and cervical esophageal injury

	Total		Thoracic		Cervical		
	(*n* = 944)		(*n* = 613)		(*n* = 331)		*p*
Mortality	113	(12.0)	86	(14.0)	27	(8.2)	0.008
1-day mortality	71	(7.5)	57	(9.3)	14	(4.2)	0.005
30-day mortality	109	(11.5)	83	(13.5)	26	(7.9)	0.009
Mechanical ventilation (days)^a^, median (IQR)	5	(2–14)	6	(2–15)	4	(2–11)	0.124
ICU stay (days)^a^, median (IQR)	7	(3–15)	7	(3–16)	6	(3–13)	0.157
Hospital length of stay (days)^a^, median (IQR)	12	(5–23)	13	(5–25)	11	(5–22)	0.131
Complications^b^							
Acute kidney injury	19	(2.5)	12	(2.5)	7	(2.5)	0.998
ARDS	43	(5.7)	31	(6.5)	12	(4.3)	0.21
Deep SSI	19	(2.5)	14	(2.9)	5	(1.8)	0.334
Pneumonia	61	(8.1)	45	(9.5)	16	(5.8)	0.072
DVT	27	(3.6)	20	(4.2)	7	(2.5)	0.23
Sepsis	26	(3.4)	23	(4.8)	3	(1.1)	0.006
PE	12	(1.6)	12	(2.5)	0	(0.0)	0.008
Cardiac arrest	16	(2.1)	10	(2.1)	6	(2.2)	0.958
Organ/space SSI	17	(2.3)	12	(2.5)	5	(1.8)	0.519
Stroke/CVA	8	(1.1)	6	(1.3)	2	(0.7)	0.717
Superficial SSI	20	(2.7)	15	(3.2)	5	(1.8)	0.265
UTI	25	(3.3)	16	(3.4)	9	(3.2)	0.927
Catheter related Blood infection	3	(0.4)	2	(0.4)	1	(0.4)	1
Overall complication	184	(24.4)	129	(27.1)	55	(19.8)	0.024
Overall infectious complication	127	(16.8)	88	(18.5)	39	(14.0)	0.115

Values are presented as median (IQR) and *n* (%)

*ICU* intensive care unit, *ARDS* acute respiratory distress syndrome, *SSI* surgical site infection, *DVT* deep vein thrombosis, *PE* pulmonary embolism, *CVA* cerebrovascular accident, *UTI* urinary tract infection, *IQR* interquartile range

^a^Include only patients without mortality (*n* = 831)

^b^Include only patients with hospital length of stay >2 days (*n* = 754)

Forward stepwise logistic regression analysis identified thoracic injury, age >50 years old, high-grade esophageal rupture (OIS IV–V), hypotension on admission, GCS <9, and severe head injury (AIS ≥3) as independent factors associated with increased mortality (Table 5). Treatment within the first 24 h was found to be a protective factor for mortality (OR 0.284; 95% CI, 0.148–0.546; *p* < 0.001) (Table 5). Injury to the thoracic esophagus with open perforation into the mediastinum was found to be an independent risk factor associated with an increased overall complication rate (OR 1.637; 95% CI, 1.06–2.53; *p* = 0.026).

**Table 5:** Independent risk factors for mortality

	Mortality				
	Adjusted p	OR	95% CI for OR		
Age >50 year	0.032	1.686	(1.045	-	2.723)
OIS I-II	Reference	Reference			
OIS III	0.578	1.151	(0.701	-	1.888)
OIS IV-V	0.03	2.256	(1.081	-	4.709)
Severe head injury (AIS ≥3)	<0.001	2.839	(1.794	-	4.493)
Thoracic injury	0.028	1.757	(1.062	-	2.907)
GCS score <9	<0.001	3.553	(2.247	-	5.618)
Hypotension	<0.001	6.087	(3.475	-	10.659)
Early treatment (≤24h)	<0.001	0.284	(0.148	-	0.546)

Logistic regression was performed with potentially causative variables (in gray) in which p value was <0.2 in univariate analysis. Multicollinearity test was checked before doing multivariate analysis

Hosmer-Lemeshow Goodness-of-Fit Test p=0.326, Cox & Snell R2=0.153, Nagelkerke R2=0.294

AUC=0.829 (95% CI=0.786-0.871, p<0.001)

*OR* Odds Ratio, *CI* Confidence Interval

## Discussion

The purpose of this study was to examine a large contemporary experience with traumatic esophageal injury, specifically with regard to the management, outcomes, and risk factors for mortality. Injury to the thoracic segment of the esophagus was found to be a major risk factor for mortality. Early treatment, within 24 h from admission, was independently associated with improved survival.

Traumatic esophageal injury is rare and associated with high morbidity and mortality. While previous studies have tried to describe outcomes, management, and risk factors for mortality, the limited sample size remained a major weakness.

In our study of more than 900 cases, the overall mortality rate was 12%. This is slightly lower compared to a 2001 retrospective multicenter study that analyzed patients with a penetrating esophageal injury (19%) [9]. This finding likely reflects recent improvements in the treatment and critical care management of such patients. In accordance with a retrospective 2013 database study of 227 patients who sustained a penetrating esophageal injury, the majority of deaths (62.8%) occurred in the first 24 h of admission due to the severity of associated injuries [10]. In our study, the mortality rate for cervical injuries was significantly lower than thoracic injuries. This result is in keeping with the current data showing that cervical injuries are associated with lower mortality [11]. This may be due to the protected anatomical location of the cervical esophagus which limits lateral bacterial spillage avoiding downward mediastinal contamination [12]. In contrast, injury to the thoracic segment of the esophagus is often associated with extensive, non-contained bacterial spillage with mediastinitis, pleural effusion, empyema, systemic sepsis, and multi-organ failure [13]. Moreover, the negative intrathoracic pressure can exacerbate the bacterial spillage from the esophageal lumen into the thoracic cavity [14].

Primary repair was the most commonly adopted surgical approach. Debridement of necrotic tissue, complete exposure of the mucosal layer, and a tension-free repair is recommended whenever feasible. Massive destructive injuries may require a more aggressive approach using esophageal resection or diversion [11]. In our study, the more invasive surgical procedures were performed for extensive esophageal injury. An early surgical procedure was performed in 79.7% of patients who underwent an operation. It has been previously advocated that early treatment is associated with improved outcomes because of limited bacterial spillage and less systemic inflammatory response. Brinster et al. in a 2004 literature review on 559 patients with esophageal perforation found that a treatment delay greater than 24 h can result in a doubled risk of mortality [7]. Similarly, Asensio et al., in a retrospective multicenter study, found that a treatment delay of greater than 13 h was associated with a significant increase in the overall complication rate and worse outcomes [9]. Discordant results were reported in a small retrospective single center study of 119 patients who sustained an iatrogenic or spontaneous perforation, with no difference in terms of mortality when comparing early and late treatment [15].

Non-operative management has been advocated for selected patients in the setting of iatrogenic and spontaneous esophageal perforation [16]. Markar et al. in a large 12-year retrospective multicenter study of 2564 patients demonstrated a significant reduction in the overall number of surgical procedures with a concomitant increase in non-operative management [17]. Minimally invasive endoscopic stenting or clipping for iatrogenic perforation has also been proposed for small discontinuities with viable, non-necrotic edges [18, 19]. These strategies may be useful in selected, hemodynamically stable trauma patients with a contained leak [20].

Because of the rarity of traumatic esophageal injury, limited data is available. For this reason, we chose to use the NTDB databank to collect a large study population, reducing the risk of a type II error. Exclusion of iatrogenic and spontaneous perforation makes our study population homogeneous, focusing only on traumatic esophageal injury. The major weaknesses of our study are related to its retrospective nature and to the fact that treatment delay of the esophageal injury may have been due to prioritizing treatment of other life-threatening injuries. We were not able to analyze the patient status in detail, and the elapsed time from the onset of symptoms to treatment was also unavailable in this administrative database. Moreover, the lack of specific details regarding the surgical procedure was a limitation.

## Conclusions

Despite improvements in surgical technique and critical care support, the overall mortality for traumatic esophageal injury remains high. The presence of a thoracic injury and extensive esophageal damage are the major independent risk factors for mortality. Early surgical treatment is associated with improved survival.
